# Health Economic Assessment: Cost-Effectiveness Thresholds and Other Decision Criteria

**DOI:** 10.3390/ijerph7041835

**Published:** 2010-04-20

**Authors:** Steven Simoens

**Affiliations:** Research Centre for Pharmaceutical Care and Pharmaco-economics, Faculty of Pharmaceutical Sciences, Katholieke Universiteit Leuven. Onderwijs en Navorsing 2, P.O. Box 521, Herestraat 49, 3000 Leuven, Belgium

**Keywords:** health economics, economic evaluation, cost-effectiveness, cost-effectiveness threshold, opportunity cost, decision criteria, multi-criteria decision analysis

## Abstract

An article published in this Journal argued that New Zealand does not apply a cost-effectiveness threshold because medicines are funded within a fixed budget and because cost-effectiveness is only one of nine criteria that inform decisions. This Comment has explained that, from a theoretical perspective, the cost-effectiveness threshold model is not inconsistent with these two arguments. The observed annual variation in incremental cost-effectiveness ratios in New Zealand may originate from yearly differences in new medicines that request reimbursement and in the budget size, and from the fact that decision makers take into account other decision criteria in addition to cost-effectiveness.

## 1. Introduction

Economic evaluation is an instrument that assesses the value of a medicine by comparing the costs and outcomes of a medicine with those of a relevant comparator. Evidence derived from economic evaluations is used to inform pharmaceutical reimbursement (and/or pricing) decisions in many countries (see Table 1). The requirement for economic evaluation fits within an overall trend towards evidence-based decision making in health care [1].

The results of an economic evaluation can be expressed in the form of an incremental costeffectiveness ratio (ICER). This ratio relates the difference in costs between a medicine and the comparator to the difference in outcomes. The ICER is then compared with a threshold ICER, which reflects the maximum cost per unit of outcome that a health care payer is willing to pay for a medicine. This means that a medicine with an ICER below the threshold value is likely to be accepted by a health care payer and a medicine with a ratio exceeding the threshold is likely to be refused.

In a previous article, I provided an overview of threshold ICERs in selected countries [2]. In a comment on this article, Metcalfe and Grocott argued that New Zealand’s Pharmaceutical Management Agency (PHARMAC) does not apply a threshold ICER for two reasons: first, New Zealand funds medicines within a fixed budget; and second, cost-effectiveness is only one of nine decision criteria used to inform decisions [3]. In the following sections, I comment on these two arguments.

## 2. Cost-Effectiveness Thresholds and a Fixed Budget

### 2.1. The Threshold ICER Model

The use of the ICER in informing decisions was originally proposed by Weinstein and Zeckhauser in 1973 [4]. These authors considered the case of a health care payer who could fund some, but not all medicines due to a budget constraint. It can be shown that, if medicines are ranked from the lowest to the highest ICER (in a so-called league table), health can be maximised by selecting medicines with increasing ICERs until the budget is exhausted. The ICER of the last medicine (*i.e.*, the medicine with the highest cost per unit of outcome ratio) to be selected is called the threshold ICER.

The threshold ICER model draws on several assumptions, including the existence of a fixed budget, the use of health maximisation as the only criterion informing resource allocation decisions, the availability of complete information on costs and outcomes of all medicines and comparators in the league table, perfect divisibility and constant returns to scale of medicines. A fixed budget means that the size of the budget does not change within a specific time period. Perfect divisibility means that a health care payer can fund medicines in infinitely small units (thus avoiding the problem of indivisibilities). Constant returns to scale apply if the ICER does not depend on the volume of medicines.

Under this model, the size of the budget determines the value of the threshold ICER. If the budget increases, medicines with higher ICERs can be selected. If the budget decreases, only the medicines with the highest value for money (*i.e.*, the lowest ICERs) can be selected. Therefore, as New Zealand funds medicines within a fixed budget, the corresponding threshold ICER can be calculated. Metcalfe and Grocott argued that the annual variation in ICERs for PHARMAC’s investments over nine years (from NZ$−40,000 to over NZ$+200,000 per quality-adjusted life year (QALY)) provides evidence that New Zealand does not apply a threshold ICER [3]. This evidence is not inconsistent with the threshold ICER model: as the availability of new medicines that request reimbursement and the size of the budget differ from year to year, the observed annual variation in ICERs in New Zealand is to be expected.

Indeed, if a country applies a fixed budget, it follows that the value of the threshold ICER is likely to change over time. For instance, new medicines constantly enter the market and old medicines are withdrawn from the market. This implies that the medicines listed in a league table change over time, thus resulting in a new value for the threshold ICER. Similarly, if the productivity in the health care sector increases through, for example, substitution of generic medicines for originator medicines, a greater health improvement can be gained from the same budget. This implies that, if the size of the budget remains constant, then the value of the threshold ICER will decrease.

### 2.2. Threshold ICERs and Opportunity Costs

Metcalfe and Grocott correctly state that threshold ICERs do not explicitly consider opportunity costs (*i.e.*, the health benefits forgone by choosing not to spend finite resources on alternatives), as they consider interventions in isolation to other potential investments [3]. Therefore, the replacement model has been proposed as an alternative to the threshold ICER model [5]. In order to assess the value of a new medicine A, the replacement model identifies an existing medicine B which, if cancelled, would generate at least enough resources to fund the incremental costs of medicine A. If the incremental outcomes associated with medicine A exceed the outcomes foregone from cancelling medicine B, then the health care payer can replace B with A, thereby increasing total health at the same or lower cost.

The advantages of the replacement model are that the assumptions underlying the threshold ICER model are not required for this decision rule. However, this model assumes that it is possible to identify a medicine B so that the resources freed up from cancelling B equal or exceed the additional costs of medicine A. Also, decision makers may find it difficult to discontinue paying for the medicine B that is replaced by the medicine A. Furthermore, the medicine B may not be the highest value alternative. Therefore, the replacement model enhances resource allocation decisions, but does not necessarily maximise population health subject to a budget constraint.

## 3. Cost-Effectiveness Thresholds and Other Decision Criteria

### 3.1. The Variable Threshold ICER Model

I agree with Metcalfe and Grocott [3] that the specification of a threshold ICER does not take into account the fact that decision makers may wish to account for other criteria in addition to costeffectiveness when assessing a medicine pricing/reimbursement decision. For instance, decision makers may wish to maximise population health subject to a budget, to reduce health inequalities in the population, to prioritise preventive over curative care, and to attach more importance to life-threatening diseases. Therefore, decision makers may not wish to apply a fixed threshold ICER, but vary the threshold ICER according to the type of medicine, the type of disease, and the decision-making context.

This may be the case for orphan medicines, *i.e.*, medicines intended for the diagnosis, prevention or treatment of a life-threatening or chronically debilitating rare disease [6]. Given their high price for an often modest effectiveness, orphan medicines are unlikely to provide value if their cost-effectiveness ratio is compared to a fixed threshold value. However, other societal considerations may matter when evaluating an orphan medicine, such as the fact that these medicines tend to target life-threatening rare diseases for which there is no alternative therapy, and that these medicines have a considerable impact on patients’ health care expenditures if they would have to incur the medicine costs themselves. The question arises as to how these various considerations can be aggregated. In other words, how can the often high cost-effectiveness ratio, weak clinical data, small health benefit, high cost and absence of an alternative therapy for orphan medicines be taken into account in a health care payer’s decision to cover such a medicine [7]? It has been argued that the value of the threshold ICER should be higher for medicines to which society attaches a high social value [8]. Orphan medicines may attract a high social value, although future research needs to elicit social values ascribed to various medicines and health technologies.

The observed annual variation in ICERs for PHARMAC’s investments [3] is not inconsistent with a variable threshold ICER model, where decision makers take into account other decision criteria in addition to cost-effectiveness.

The variable threshold ICER model is applied in a number of countries. For instance, the Scottish Medicines Consortium has assessed the value of over 200 medicines from 2002 to 2005, including 39 cancer medicines [9]. Of these cancer medicines, 11 medicines were accepted, 15 medicines were accepted with restrictions, and 13 medicines were not recommended. A review of the 200 applications showed that fewer randomized controlled trials were available for cancer medicines and that cancer trials had a longer follow-up period than other medicines. There was evidence that a higher threshold ICER may apply to cancer medicines: although cancer medicines had a higher cost per QALY than non-cancer medicines (median of £15,000 (around US$24,309) per QALY *versus* £8,500 (around US$13,775) per QALY), acceptance rates were similar (66.7% *versus* 66.4%).

### 3.2. Multi-Criteria Decision Analysis

The fact that PHARMAC considers nine decision criteria to inform decisions can be seen as an application of multi-criteria decision analysis. According to multi-criteria decision analysis [10], an expert panel defines the relevant decision-making criteria and their relative importance. Each criterion needs to be measurable, so that the degree to which a medicine attains the criterion can be assessed. Different techniques to aggregate the scores of a medicine on the different criteria can be applied to calculate the overall performance of a medicine. It follows that decision makers allocate resources based on the ranking of medicines according to their performance scores until the budget is exhausted.

## 4. Conclusions

This Comment has argued that, from a theoretical perspective, the threshold ICER model is not inconsistent with the fact that New Zealand funds medicines within a fixed budget and that costeffectiveness is only one of nine decision criteria used to inform decisions. The observed annual variation in ICERs in New Zealand may originate from yearly differences in the availability of new medicines that request reimbursement and the size of the budget, and from the fact that decision makers take into account other decision criteria in addition to cost-effectiveness.

## Figures and Tables

**Table 1.** Use of economic evaluation in decision making around the world.

*Country*	*Organisation*	*Implementation date*
Australia	Pharmaceutical Benefits Advisory Committee	1993
Belgium	Medicine Reimbursement Committee	2002
England/Wales	National Institute for Health and Clinical Excellence	1999
France	High Health Authority	2008
Germany	Institute for Quality and Efficiency in Health Care	2007
Netherlands	Health Care Insurance Board	1999
New Zealand	Pharmaceutical Management Agency	1993
Scotland	Scottish Medicines Consortium	2002
Sweden	Dental and Pharmaceutical Benefits Agency	2002
Taiwan	Centre for Medicine Evaluation	2008

## References

[1] Perleth M Jakubowski E Busse R What is ‘best practice’ in health care? State of the art and perspectives in improving the effectiveness and efficiency of the European health care systems Health Policy 2001 56 235 250 11399348 10.1016/s0168-8510(00)00138-x

[2] Simoens S Health economic assessment: a methodological primer Int. J. Environ. Res. Public Health 2009 6 2950 2966 20049237 10.3390/ijerph6122950 PMC2800325

[3] Metcalfe S Grocott R Health economic assessment: New Zealand in fact has no costeffectiveness threshold Int J Environ Res Public Health 2010in press 10.3390/ijerph7041831 PMC2872338 20617061

[4] Weinstein MC Zeckhauser R Critical ratios and efficient allocation J. Public Econ 1973 2 147 157

[5] Sendi P Gafni A Birch S Opportunity costs and uncertainty in the economic evaluation of health care interventions Health Econ 2002 11 23 31 11788979 10.1002/hec.641

[6] European Commission Regulation (EC) No 141/2000 of the European Parliament and the Council of 16 December 1999 on orphan medicinal products Official Journal of the European Communities 2000 L 18/1

[7] Denis A Simoens S Fostier C Mergaert L Cleemput I Health Technology Assessment Policy Governing Orphan Diseases and Orphan Medicines Belgian Health Care Knowledge Centre Brussels, Belgium 2009

[8] Drummond M Evans B LeLorier J Karakiewicz P Martin D Tugwell P MacLeod S Evidence and values: requirements for public reimbursement of drugs for rare disease—a case study in oncology Can. J. Clin. Pharmacol 2009 16 e273 e281 19439771

[9] Timoney A Walker A Paterson K Bennie M McIver L Webb D The Scottish Medicine Consortium—are oncology medicines different? Presentation at the Third Health Technology Assessment International Conference Adelaide, Australia 2006

[10] Baltussen R Niessen L Priority setting of health interventions: the need for multi-criteria decision analysis Cost. Eff. Resour. Alloc 2006 4 14 16923181 10.1186/1478-7547-4-14 PMC1560167

